# Safety of indocyanine green for lymph node mapping in early-stage vulvar cancer: multicenter evaluation and systematic review

**DOI:** 10.1007/s00404-025-08244-8

**Published:** 2026-01-14

**Authors:** Alberto Rafael Guijarro-Campillo, Pablo Padilla-Iserte, Bárbara Couso, Diego Erasun, Jesús Utrilla-Layna, Octavio Arencibia, Juan Gilabert-Estellés, Nadia Veiga, Ana Boldo-Roda, Víctor Lago, Anibal Nieto, Santiago Domingo

**Affiliations:** 1 Department of Gynecologic Oncology, Hospital Clinico Universitario Virgen de la Arrixaca, Murcia, Spain; 2 University of Valencia, Valencia, Spain; 3 Department of Gynecologic Oncology, University and Polytechnic Hospital of La Fe, Valencia, Spain; 4 Department of Obstetrics and Gynecology, University Hospital of Ourense, Ourense, Spain; 5 Department of Obstetrics and Gynecology, Hospital Marqués de Valdecilla, Santander, Spain; 6 Department of Gynecological Oncology, Fundación Jiménez Díaz, Madrid, Spain; 7 Department of Gynecologic Oncology, Complejo Hospitalario Universitario Insular Materno Infantil, Las Palmas de Gran Canaria, Spain; 8 Department of Obstetrics and Gynecology, University General Hospital of Valencia, University of Valencia, Valencia, Spain; 9 Department of Gynecologic Oncology, Complejo Hospitalario de Navarra, Pamplona, Spain; 10 Department of Obstetrics and Gynecology, University Hospital La Plana, Castellón, Spain; 11 San Pablo University, CEU, Madrid, Spain; 12 Madrid, Spain

**Keywords:** Indocyanine green, Sentinel lymph node biopsy, Vulvar cancer, Allergic reactions

## Abstract

**Purpose:**

This study aimed to evaluate the rate and severity of allergic events associated with the use of indocyanine green (ICG) in sentinel lymph node biopsy (SLNB) for patients with early-stage vulvar cancer. The research question focused on whether ICG administration poses a significant risk of allergic reactions, especially in patients with a history of allergies to iodinated contrast.

**Methods:**

We conducted a retrospective study after prospective multicenter recruitment endorsed by the Spanish Investigational Network Gynecologic Oncology Group. Data on patient demographics, history of allergic reactions, and ICG administration were collected. A systematic literature review was performed to assess existing studies on ICG-related allergic reactions in SLNB.

**Results:**

Among the 75 patients, 66 (75%) have been exposed to iodinated contrasts and 2 (3%) had a documented iodinated contrast allergy with a mild reaction. There were no intraoperative complications. During the postoperative period, there were only minor complications (15 (20.0%) grade I and 13 (17.3%) grade II of Clavien–Dindo classification), and none associated with the injection of ICG or allergen-based that could be related. The systematic review of 11 studies involved 206 patients and 354 groins. The history of allergy to iodinated contrast was not recorded in any of them. We observed no patients with adverse reactions related to this tracer after peritumoral injection.

**Conclusion:**

Our findings suggest that peritumoral ICG injection for SLNB in early-stage vulvar cancer could be safe. This study reinforces the potential for ICG to be a reliable tracer in vulvar cancer staging.

## What does this study add to the clinical work?

**Table:** 

This study demonstrates that peritumoral injection of indocyanine green for sentinel lymph node mapping in early-stage vulvar cancer is safe, even in patients with a history of allergies to iodinated contrast, thereby reinforcing its potential as a reliable tracer in clinical practice.	

## Introduction

Surgery is the primary treatment for early-stage vulvar cancer, including radical local excision with groin evaluation [1, 2]. Vulvar cancer guidelines recommend inguinal sentinel lymph node biopsy (SLNB) as the standard of care for patients with unifocal squamous tumors < 4 cm and clinically non-suspicious nodes in the groin [3, 4]. Recently, some studies have demonstrated the feasibility and accuracy of SLNB using indocyanine green (ICG), with promising results [5, 6].

ICG is a water-soluble green dye that consists of about 5% sodium iodide. Once it enters the bloodstream, it quickly binds to more than 95% of plasma proteins and is eliminated through bile [7]. The metabolism of ICG relies solely on a specific transport system in the liver. Its maximum fluorescent emission occurs at 835 nm, which is optimal for near-infrared (NIR) applications [8]. Although ICG has been used intravenously for decades in various medical fields (cardiology, ophthalmology, hepatology, neurosurgery) [9], its use in gynecologic oncology is more recent. This dye has received approval from the U.S. Food and Drug Administration for intravenous injection. Despite the rapid and consistent expansion of its use for sentinel lymph node mapping, data on the risk of allergic reactions related to ICG remain scarce. A single study published with this objective in the case of endometrial cancer, and extrapolated data from cervical cancer trials, showed less than 1% of mild to moderate reactions after intracervical injection. In fact, interstitial and cutaneous injections for lymphatic mapping, as in vulvar cancer, are still not among its factory indications [10].

Iodinated contrast allergies have traditionally been considered a contraindication to the use of ICG due to its sodium iodide component. As such, patients with reported allergies to iodine or iodinated contrast were excluded in several of the major ICG-SLNB vulvar cancer trials [11–14]. Despite this theoretical risk, there are limited data on the true risk of cross-reactivity between iodinated contrast and ICG. There is also a prevailing belief that patients with a history of shellfish allergies or similar allergic reactions to contrast agents should not receive indocyanine green or should be pre-medicated before administration; however, no known evidence supports this.

Current data on adverse reactions in these specific groups are insufficient for a comprehensive risk assessment. Therefore, investigating the adverse effects of ICG in diverse patient populations allows for better identification of high-risk groups and the proposal of effective preventive measures. The main objective of this study is to evaluate the rate and severity of allergic events in all patients with vulvar cancer undergoing surgical staging in our previously published series, with intraoperative peritumoral injection of indocyanine green for sentinel lymph node mapping [6]. We also add a systematic literature review regarding ICG administration in SLNB for vulvar cancer to explore allergies or adverse reactions. The justification for the study is represented by the potential to strengthen the evidence supporting the safety of indocyanine green when administered to patients with early-stage vulvar cancer at the peritumoral level in a large and heterogeneous population with varying allergic profiles.

## Methods

### Multicenter observational study

**Fig. 1:**
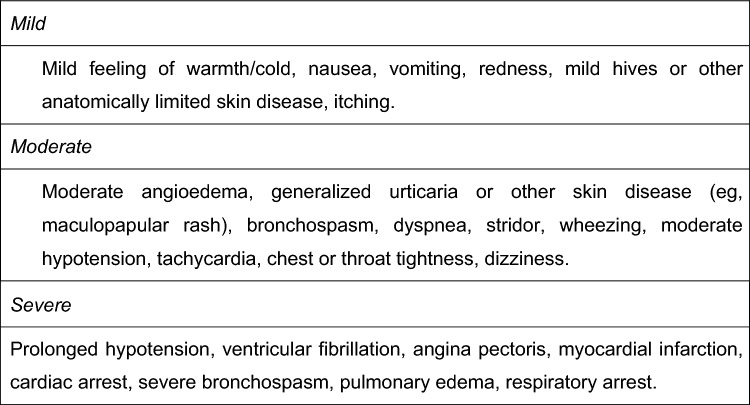
Severity grading system for acute allergic reactions [18]

**Fig. 2:**
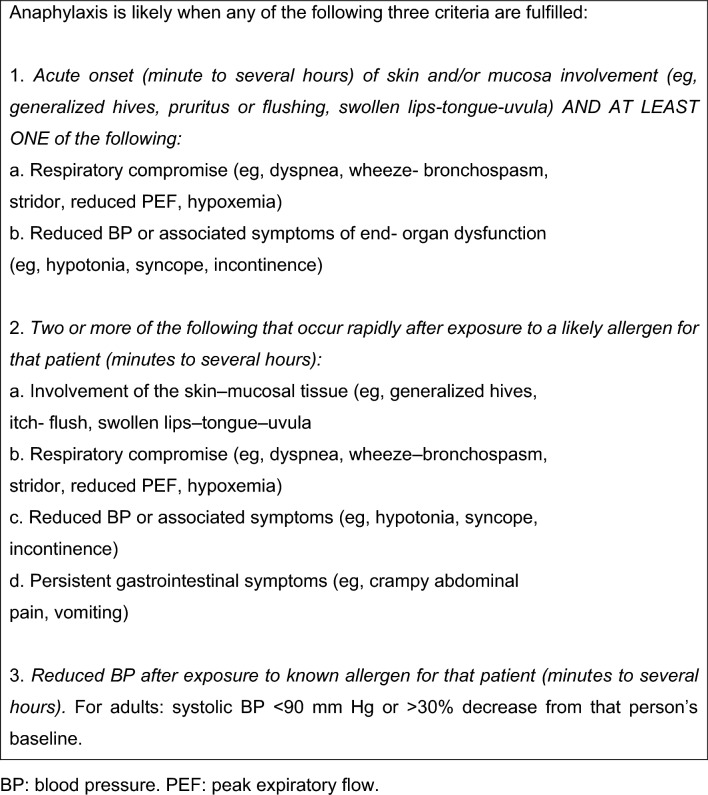
European Academy of Allergy and Clinical Immunology 2021 and Sampson clinical criteria for the diagnosis of anaphylaxis [18, 19]

This retrospective study after prospective multicenter recruitment was endorsed by the Spanish Investigational Network Gynecologic Oncology Group (Spain-GOG). It was conducted after Institutional Review Board approval in eight centers, from June 2018 to January 2022. It was performed in accordance with the Helsinki Declaration of Ethical Principles. All patients provided written consent. All study researchers complied with data confidentiality in accordance with General Data Protection Regulations [15]. Patients were eligible if they met SLN criteria: unifocal tumors, squamous-cell carcinoma with stromal invasion > 1 mm, tumor size < 4 cm, and clinically and radiologically negative groin areas [16]. Patients with non-squamous histology, tumor size ≥ 4 cm, multifocal tumors, suspected lymph node involvement, and those who were unfit for standard surgery were excluded. Tracer doses were calculated according to current recommendations for SLN procedures [17]. Thus, before beginning surgery and after anesthesia, a 25 mg vial of ICG (Pulsion Medical System, Munich, Germany) was dissolved in 10 mL of sterile water (2.5 mg/mL): 2 mL was injected in 4 intradermal quadrants around the tumor (0.5 mL per injection). This technique was applied by all centers in a standardized way. The electronic medical record was used to abstract data regarding patient demographics: age, body mass index (BMI), history of non-drug-associated allergic reactions, history and severity of drug-associated allergic reactions, history and severity of allergy to contrast media, and liver and kidney pathology. The ICG re-injection, intraoperative, and postoperative data associated with allergic reactions related to the use of ICG, the time until these reactions occurred, their severity, and instances of anaphylaxis were analyzed. The patient after surgery followed a similar visit protocol in all centers at 2 and 4 weeks and then every 3 months. The patient was considered out of the study when 3 months of follow-up were reached. An early complication was defined as one that occurred before 30 days postoperatively. A contact was provided to notify any adverse event and possible assessment urgently. The severity of allergic reactions and anaphylaxis were defined according to the European Academy of Allergy and Clinical Immunology 2021 guidelines and the Sampson criteria 2006 (Figs. 1 and 2) [18, 19]. To estimate the 95% confidence interval for the incidence of allergic reactions in our sample, the Wilson method was used. This method is particularly suitable for situations where zero events are observed in the sample, as it provides a more robust approach for calculating the confidence interval compared to traditional methods.

### Systematic review

#### Literature search

The protocol of the study was registered on PROSPERO CRD (CRD420251088749) on July 6, 2025. We report our systematic review based on the recommendation of the Preferred Reporting Items for Systematic Reviews and Meta-Analyses (PRISMA) 2020 Guideline. Observational and randomized controlled trials were included. The following population/intervention/control/outcome (PICO) approach was employed. However, this study is based on a single-arm analysis, without an active comparator, evaluating the safety of ICG in patients with vulvar cancer:

P—patients diagnosed with vulvar cancer undergoing SLNB.

I—ICG

C—an active comparator was not used; this is a single-arm analysis.

O—primary outcome: allergic reaction associated with the use of ICG.

Literature search was managed in: Medline, Embase, Cochrane, Web of Science and Scopus, from inception to June 2025. Search strategy: The key search terms included: vulv*, sentinel, indocyanine green. We used combinations of these terms in “and/or”. Only articles published in English or available in English translation were included. Studies were also excluded if the dose of ICG was not reported and if the reactions associated with the injections of tracers were not recorded as a variable. Accompanying a statistical meta-analysis was considered inappropriate due to the lack of randomized controlled trials, differences in concentrations and administration methods of ICG, and varying protocols for its use.

#### Data collection

**Fig. 3:**
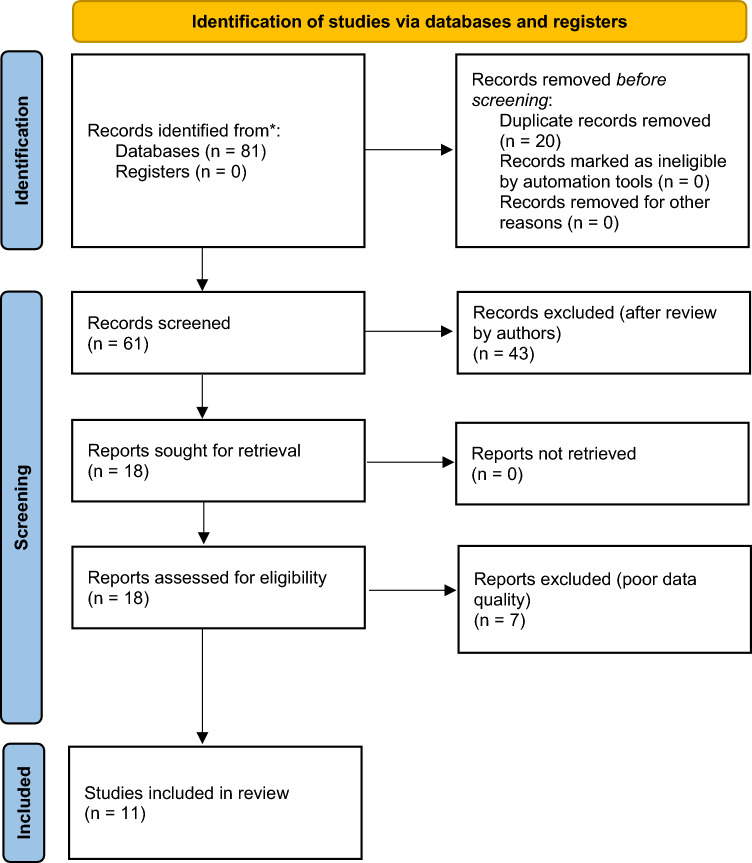
PRISMA 2020 flow diagram of the study selection process

After a systematic search of the databases, duplicate removal and selection were made according to the PICO criteria, two independent authors (A.R.G and P.P) screened the publications separately for title and abstract. After removing duplicates, we screened 61 articles, 18 articles were excluded during the title and abstract selection, and another 25 articles were excluded during the full-text selection. After full-text selection, a further 7 articles were excluded due to poor data quality, leaving 11 articles selected for the systematic review (Fig. 3). Any disagreements were resolved by a third reviewer (V.L). Of the 11 studies, a total of 206 patients were included for analysis [11, 12, 14, 17, 20–28]

We collected the following data from eligible articles: first author, year of publication, study type, study design, details of treatments received, patients with history of non-drug-associated allergic reactions, history and grade of drug-associated allergic reactions, history and grade of allergy to contrasts media, liver and kidney pathology and intraoperative and postoperative data related with allergic reactions associated with the use of ICG and the time until the same.

#### Quality assessment

The risk-of-bias assessment in the outcomes was carried out using Risk of Bias 2, a revised risk-of-bias tool for randomized trials, and Risk Of Bias In Non-randomized Studies—of Interventions (ROBINS-I), a tool for assessing the risk of bias in nonrandomized studies [23].

## Results

### Multicenter observational study

A total of 75 patients with vulvar cancer who complete surgery and received peritumoral indocyanine green injection for SLNB were retrospectively reviewed. Patient characteristics are shown in Table 1. Thirty-eight had lateral and thirty-seven had midline tumors. The mean age was 67.49 ± 13.67 (range 35–88) and the mean BMI was 27.77 ± 5.80 (range 18–47). Most (61.3%) had an ECOG performance status of 0, with a Charlson Comorbidity Index mean of 5.04 ± 2.08 (range 2–11). A total of 21, 28% had a history of drug allergic reactions, of whom only 1 (1.3%) had an episode of severe reaction to pyrazolines. Drug categories included mostly antibiotics (13, 17.3%). Most patients have been exposed to iodinated contrasts (66, 75%), only two (2.9%, 95% CI 0–7.4) had a documented iodinated contrast allergy with a mild reaction in both cases. Among these patients with an iodinated contrast allergy, none received corticosteroids with or diphenhydramine prior to ICG injection.

**Table 1:** Characteristics, surgical and postoperative data of 75 patients who underwent ICG-SLNB

Variable	ICG-SLNB patients (N = 75)
Age mean ± SD (range)—years*	67 ± 13 (35–88)
BMI mean ± SD (range)—kg/m^2^*	27.77 ± 5.80 (18–47)
ECOG 0 n (%)	46 (61.3%)
CCI mean ± SD (range)*	5.04 ± 2.08 (2–11)
History of allergies	
History of non-drug allergic reactions n (%)	5 (6.7%)
Hives	1 (1.3%)
Asthma	1 (1.3%)
Conjunctivitis	2 (2.6%)
Rhinitis	1 (1.3%)
History of drug allergic reactions n (%) ^	21 (28%)
Acetylsalicylic acid	2 (2.6%)
Pyrazolines	5 (5.3%)
Penicillin and derivatives	6 (8%)
Sulfonamide	2 (2.6%)
NSAIDs	1 (1.3%)
Clavulanic acid	1 (1.3%)
Tramadol (opioid)	1 (1.3%)
Acetylcysteine	1 (1.3%)
Severity of drug allergic reactions n (%)	
Mild	18 (24%)
Moderate	2 (2.6%)
Severe	1 (1.3%)
Previous exposition iodinates contrast media n (%)	66 (88%)
Previous reaction iodinates contrast media n (%)	2 (2.6%)
Severity of iodinates contrast media reactions n (%)	
Mild	2 (2.6%)
History of kidney/liver disease	
Chronic moderate/severe liver disease n (%)	0 (0%)
Chronic moderate/severe kidney disease n (%)	4 (5.3%)
Surgical data	
Intraoperative complications	0 (0%)
Intraoperative reactions ICG associated n (%)	0 (0%)
Time from ICG injection	NA
ICG re-injection n (%)	0 (0%)
ICG–SLN detection	
Unilateral (1 SLN)	36 (48%)
Unilateral (≥ 2 SLNs)	10 (13.3%)
Bilateral migration	16 (21.3%)
Postoperative data	
Postoperative reactions ICG associated n (%)	0 (0%)
Time from ICG injection	NA
Complications (Clavien–Dindo classification) n (%)	
I	15 (20.0%)
II	13 (17.3%)

*ICG* indocyanine green; *SLNB* sentinel lymph node biopsy; *ECOG* eastern cooperative oncology group; *CCI* Charlson comorbidity index; *NSAIDs* non-steroidal anti-inflammatory drugs

*Data were summarized using the mean (standard deviation) / n (%). ^Some of the patients may have allergies to several medications

There were no intraoperative complications. During the postoperative period, there were only minor complications (15 (20.0%) grade I and 13 (17.3%) grade II of Clavien-Dindo classification), and none associated with the injection of ICG or allergen-based that could be related (0%, 95% CI 0–4.87). No patients experienced an anaphylactic response after ICG injection (0%, 95% CI 0–4.87).

### Systematic review

All included studies have obtained informed consent from study participants and protocols approved by an ethics committee or institutional review board (Fig. 3). A total of 11 studies involving 206 patients and 354 groins were evaluated.

**Fig. 4:**
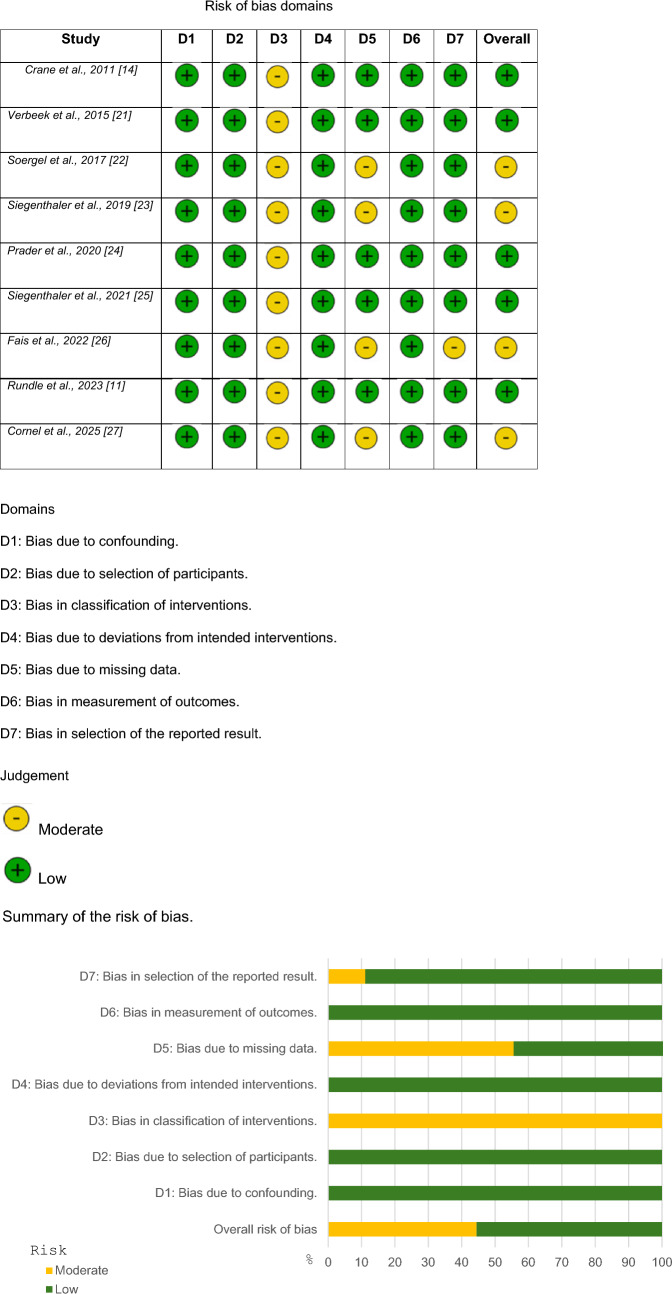
Detailed risk-of-bias chart. The ROBINS-I tool was used to evaluate the risk of bias of each study

**Fig. 5:**
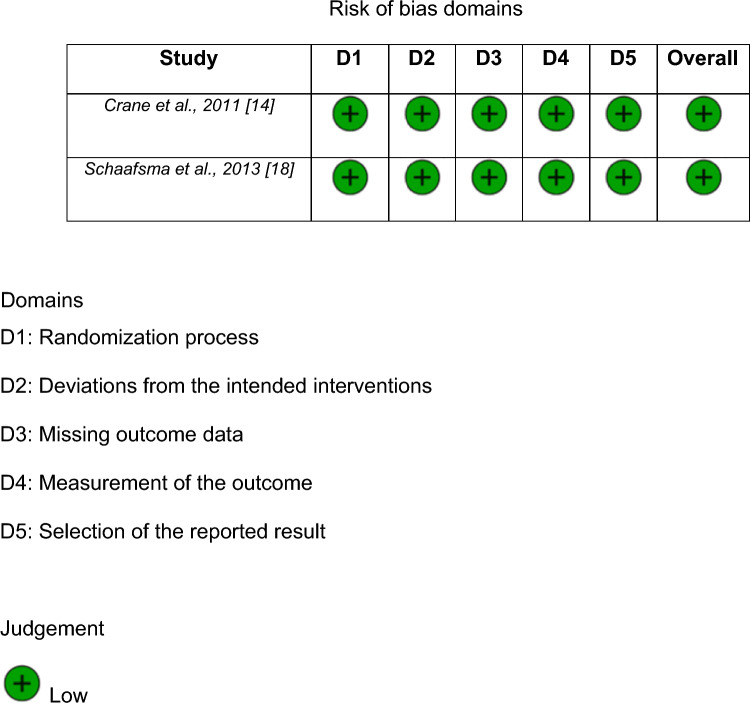
Detailed risk-of-bias chart for RCTs. The Rob2 tool was used to evaluate the risk of bias of each study

The majority of the studies are observational; therefore, they have a moderate risk of bias. However, some studies were well designed, resulting in a low risk of bias. Nine articles were observational studies and for these, the ROBINS-I tool was used to determine the risk of bias. For the two RCT studies, the Rob2 tool was used. Risk-of-bias evaluations are available in Figs. 4, 5.

We observed no patients with signs or symptoms of allergic reaction and determined that the cause of other adverse reactions was likely unrelated to this tracer peritumoral injection (0%, 95% CI 0–1.07) (Table 2).

**Table 2:** Review of the studies reporting allergic reaction after peritumoral indocyanine green injection for early-stage vulvar cancer

Author, year	Study design	Patients	Groins inspected	Patients history of iodine contrast or other allergies	ICG injection (dosage and technique)	Other tracers at same groin	Allergic reactions	Grade of allergy	Time from injection
Crane et al. [14]	P	10	16	NR	0.5 mg/mL, 0.5 mL each peritumoral	BD and 99mTc	0	NA	NA
Schaafsmaet al. [17]	P	24	34	Patients with allergy to iodine were excluded	1.6 mL total of ICG-HSA or ICG at 4 sites (1 mL each) peritumoral	BD and 99mTc	0	NA	NA
Verbeek et al. [20]	P	12	20	NR	2.5 mg/mL or ICG–99mTc 160 nmol in 1 mL (0.12 mg) at 4 sites (1 mL each) peritumoral	BD and 99mTc	0	NA	NA
Soergel et al. [21]	P	27	52	NR	2.5 mg/mL, at 4 sites (0.5 mL each) day before surgery	BD and 99mTc	0	NA	NA
Siegenthaler et al. [22]*	R	NR	5	NR	NR	99mTc	0	NA	NA
Deken et al. [12]	RCT	24	36	Patients with allergy to iodine were excluded	25 mg ICG in 5 mL sterile water. 50 μl of ICG solution (5 mg/ml) added to99mTc in 2 mL. A dose of 52–100 MBq (ICG concentration 161 μmol/l) 3–20 h before surgery	99mTc	0	NA	
Prader et al. [23]	R	33	64	NR	2.5 mg/mL, at 4 sites (0.5 mL each) peritumoral	99mTc	0	NA	NA
Siegenthaler et al. [24]	R	34	64	NR	2.5 mg/mL, at 4 sites (0.5 mL each) peritumoral	99mTc and/or BD	0	NA	NA
Fais et al. [25]*	R	2	NR	NR	NR	99mTc	0	NA	NA
Rundle et al. [11]	P			Patients with allergy to shellfish, iodine or ICG were excluded	2.5 mg/mL, at 4 sites (0.25 mL each) peritumoral	BD and 99mTc	0	NA	NA
Cornel et al. [26]	R	40	63	NR	2.5 mg/mL, at 4 sites (0.25–0.5 mL each) peritumoral	22 groins ICG alone, 41 with 99mTc and/or BD	0	NA	NA

*P* prospective; *R* retrospective; *RCT* randomized control trial; *NR* not registered; *NA* not applicable; *HSA* human serum albumin; *BD* blue dye

*Communication to congress abstract

The history of allergy to iodinated contrast was not recorded in any of them except in three, which was part of the exclusion criteria for these studies [11, 12, 17].

The concentration of ICG employed in mostly all studies was the same (1.25 mg/mL) excluding in the two studies where it was combined with 99mTc-nanocolloid [12, 20] and one with BD [14] where the concentrations of ICG were much lower.

In all cases, ICG solely or combined with nanocolloid, was injected intracutaneously at peritumoral locations, at the time of surgery or before (3–24 h previously), respectively. At least 99mTc was associated in all groins assessed with or without patent blue dye (BD) as a modality for detecting SLN. There were only 15 patients (22 groins) where ICG was used as a single detection modality .

## Discussion

This study demonstrates that, within a clinically heterogeneous population of patients with a history of allergies and exposure to contrast media undergoing sentinel lymph node biopsy for surgical staging of early-stage vulvar cancer, peritumoral injection of indocyanine green did not result in any anaphylactic or other immediate allergic reactions. Furthermore, it is unlikely to have played a role in delayed skin reactions, even in patients with iodinated contrast allergies.

A review of the existing literature indicates that no adverse reactions have been documented with vulvar peritumoral ICG injections at total volumes of 1–2 mL (1.25 mg/mL) or less.

In the context of other gynecologic tumors, the use of ICG for SLN mapping in endometrial cancer patients has an excellent safety profile, with a low incidence of allergic reactions or adverse events, as supported by three major prospective studies [29–31]. While it is true that two of these studies excluded patients with a history of allergies to iodinated contrast [29, 30], one reported a patient who presented to the emergency room on the sixth postoperative day with facial swelling. Although the late onset of this rash did not suggest a temporal relationship to the use of BD or ICG, it was deemed possible that it was related [30]. However, delayed allergic-like reactions to intravenous iodinated contrast typically manifest as cutaneous responses within 3 h to 2 days post-administration [32]. Moreover, the co-administration of BD complicates the situation as it is associated with IgE-mediated allergic reactions, whereas no such risks have been established for ICG [33]. Zamarelli et al. reported on 1,414 consecutive endometrial cancer patients, including 67 with documented iodine or contrast allergies, showing no ICG-related allergic reactions or adverse events; however, 97% of these patients received hydrocortisone, dexamethasone, or both, with or without diphenhydramine as part of an institution-wide anti-emetic protocol [34]. The only reported case of anaphylaxis following intracervical ICG administration occurred after the injection of a total of 40 mg ICG (8 mL of a 5 mg/mL ICG solution), which is significantly higher than common doses used [35]. No information was provided regarding allergy histories or whether the patient received premedication. Moreover, no allergic reactions associated with intracervical ICG during sentinel node mapping in early cervical cancer have been published to date [36]. The doses and volumes used in most published studies were 25 mg/mL and 4 mL for patients with endometrial and cervical cancer, respectively [36, 37].

There is a significant difference in injection sites that complicates comparisons between sentinel lymph node mapping for vulvar cancer and other female genital tract tumors. In vulvar cancer, the injection is peritumoral, while in endometrial and cervical cancers, it is intracervical. Breast cancer may serve as a more comparable context due to similarities in lymphatic anatomy and tumor proximity to the skin. In addition, the injection sites (intradermal/subdermal, subareolar/periareolar, or peritumoral) as well as the doses and volumes employed (< 5 mg/mL, 2 mL) are similar. Notably, no adverse events associated with fluorescein have been reported in studies involving breast cancer patients where this method was utilized for SLNB [38]. Melanoma is another cancer with similar characteristics regarding sentinel node mapping and has been analyzed alongside breast cancer on occasion [39]. In studies using similar doses (2.5 mg/mL), no associated side effects have been described in these patients [40].

Information regarding dose–intensity relationships between food allergens and anaphylaxis is well established [41]. The sole case of anaphylaxis associated with ICG in gynecological cancer involved a higher-than-usual dose, as noted earlier. Currently, there are no standardized recommendations regarding the concentration and volume of ICG during lymphatic mapping procedures for vulvar cancer [2, 3]. The concentration and volume administered in our study align with published data (1.25 mg/mL, total range of 2–4 mL). Re-injection was not considered for any of our patients [6]. The existing literature also reflects that larger tracer volumes were not employed in cases of non-migration [11, 12, 14, 17, 20–26].

Rare adverse reactions to ICG are similar to non-immunological reactions associated with intravenous contrast media. It is often mistakenly believed that these reactions are caused by IgE-mediated responses to iodine when, in fact, they arise from the release of histamine by basophils and mast cells [42]. Physiological reactions to high-osmolality contrast media can mimic cardiovascular effects of allergic reactions but are not related to IgE-mediated allergies [43]. The introduction of low-osmolality contrast media has significantly reduced these reactions, reinforcing their non-allergic cause [44]. IgE-mediated reactions are rare and not linked to iodine, just as shellfish allergies are due to proteins like tropomyosin, not iodine [45]. Confirming this, in our study, neither of the two patients with a history of iodinated contrast allergy experienced adverse reactions to ICG; however, the number of such patients is very small, and rare events cannot be excluded. In most of the included studies, there was no consideration of a history of allergy to iodinated contrasts or other substances such as shellfish; however, in three studies, these patients were excluded [11, 12, 17]. Although there have been no reported adverse reactions associated with the use of ICG, if they had occurred, it would be challenging to determine the true origin in studies that also injected BD [11, 14, 17, 20, 21, 24], since it is known that BD presents a risk of IgE-mediated allergic reactions and anaphylactic shock.

Regarding the use of ICG across medical fields, a recent study analyzed adverse events (AEs) in a larger and more diverse population by utilizing real-world data from the Food and Drug Administration Adverse Event Reporting System (FAERS) database. From 2004 to 2023, a total of 62 adverse event reports related to ICG were identified. The top 3 indications and uses of ICG were vitrectomy (*n* = 7, 11.29%), angiogram (*n* = 6, 9.68%), and imaging procedures (*n* = 4, 6.45%); however, in 23 cases (37.10%), the indication was unknown. The authors emphasize the need for vigilant monitoring and prevention in the clinical use of ICG and advocate for further research to understand the associated risks and maximize its benefits as a therapeutic or diagnostic agent [10].

### Strengths and limitations

In addition to the retrospective design, one of the main limitations of this study was its relatively small population of patients with contrast allergies and no associated adverse events, rendering it underpowered to detect rare events. In addition, the follow-up time to the event was not rigorously established, which could extend beyond 7 days [46]. Like all systematic reviews, the quality of our study and the evidence synthesized depend on the quality of the included studies. A significant weakness of our study is the heterogeneous design of the 11 studies included, with only 2 being RCTs, limited information on the history of contrast allergy, and the use of ICG in conjunction with other contrasts. All of this means that the conclusions regarding the absence of association between the use of ICG and rare adverse effects are limited.

Despite these limitations, this is the only study to date that examines this hypothesis within a large population through a systematic review of vulvar cancer patients who received ICG for sentinel lymph node mapping.

### Implications for practice and future research

The use of indocyanine green appears to be the most effective tracer, and its use is becoming increasingly widespread in conjunction with a radiotracer for sentinel lymph node mapping in vulvar cancer. Our findings contribute to the growing body of evidence on the safety of this tracer with the techniques and doses employed, thus, far. Future prospective studies should include patients with a previous history of contrast and shellfish allergies and meticulously record any allergic-type reactions and their outcomes.

There is a minimal risk of allergy to indocyanine green, and the evidence clarifies that patients with shellfish and other contrast allergies could safely receive it, enhancing vulvar cancer staging for most patients. Considering this, it may be feasible to propose the possibility of repeating the injection of indocyanine green in cases where adequate contrast migration is not achieved, within the framework of future study designs.

## Conclusion

In summary, no patients in this cohort demonstrated an adverse reaction after peritumoral injection with doses of 5 mg or less for SLN mapping in early-stage vulvar cancer. After evaluating the available literature, no adverse reactions or anaphylaxis have been proven with vulvar peritumoral ICG injection. Therefore, while a low but non-zero risk of rare reactions cannot be excluded, and as ICG has gained widespread popularity, we anticipate that more information regarding the safety of intradermal injection for SLN mapping in vulvar cancer will accumulate in the coming years.

## Data Availability

No datasets were generated or analysed during the current study.
